# The NOURISHED randomised controlled trial comparing mentalisation-based treatment for eating disorders (MBT-ED) with specialist supportive clinical management (SSCM-ED) for patients with eating disorders and symptoms of borderline personality disorder

**DOI:** 10.1186/s13063-016-1606-8

**Published:** 2016-11-17

**Authors:** Paul Robinson, Jennifer Hellier, Barbara Barrett, Daiva Barzdaitiene, Anthony Bateman, Alexandra Bogaardt, Ajay Clare, Nadia Somers, Aine O’Callaghan, Kimberley Goldsmith, Nikola Kern, Ulrike Schmidt, Sara Morando, Catherine Ouellet-Courtois, Alice Roberts, Finn Skårderud, Peter Fonagy

**Affiliations:** 1 University College London, London, UK; 2 Barnet Enfield and Haringey Mental Health Trust, St Ann’s Hospital, London, UK; 3 IOPPN, King’s College London, London, UK; 4 Kent and Medway Partnership Trust, The Red House, Maidstone, UK; 5 Anna Freud Centre, London, UK; 6 South London and the Maudsley NHS Trust, London, UK; 7 Institute for Eating Disorders, Oslo, Norway

**Keywords:** Eating disorders, Psychotherapy, Borderline personality disorder, Mentalisation-based treatment, Specialist supportive clinical management, Randomised controlled trial, Drop-out

## Abstract

**Background:**

In this multi-centre randomized controlled trial (RCT) we compared modified mentalisation-based treatment (MBT-ED) to specialist supportive clinical management (SSCM-ED) in patients with eating disorders (EDs) and borderline personality disorder symptoms (BPD). This group of patients presents complex challenges to clinical services, and a treatment which addresses their multiple problems has the potential to improve outcome. MBT has been shown to be effective in improving outcome in patients with BPD, but its use has not been reported in ED.

**Methods:**

Sixty-eight eligible participants were randomised to MBT-ED or SSCM-ED. The primary outcome measure was the global score on the Eating Disorder Examination. Secondary outcomes included measures of BPD symptoms (the Zanarini Rating Scale for Borderline Personality Disorder), general psychiatric state, quality of life and service utilisation. Participants were assessed at baseline and at 6, 12 and 18 months after randomisation. Analysis was performed using linear mixed models.

**Results:**

Only 15 participants (22 %) completed the 18 month follow-up. Early drop-out occurred significantly more in the SSCM-ED group. Drop-out did not vary with treatment model later in therapy and was sometimes attributed to participants moving away. There was higher drop--out amongst smokers and those with higher neuroticism scores. 47.1 % of participants in the MBT-ED arm and 37.1 % in the SSCM-ED arm attended at least 50 % of therapy sessions offered.

Amongst those remaining in the trial, at 12 and 18 months MBT-ED was associated with a greater reduction in Shape Concern and Weight Concern in the Eating Disorder Examination compared to SSCM-ED. At 6, 12 and 18 months there was a decline of ED and BPD symptoms in both groups combined.

Ten participants were reported as having had adverse events during the trial, mostly self-harm, and there was one death, attributed as ’unexplained’ by the coroner.

**Conclusions:**

The high drop-out rate made interpretation of the results difficult. Greater involvement of research staff in clinical management might have improved compliance with both therapy and research assessment. MBT-ED may have had an impact on core body image psychopathology.

**Trial registration:**

Current Controlled Trials: ISRCTN51304415. Registered on 19 April 2011.

**Electronic supplementary material:**

The online version of this article (doi:10.1186/s13063-016-1606-8) contains supplementary material, which is available to authorized users.

## Background

Patients with eating disorders (EDs) and co-occurring symptoms of borderline personality disorder (BPD) present considerable clinical challenges. Their eating disorder is associated with self-harm, impulsivity, physical problems, substance abuse and ambivalence about treatment. Their complex natural history, with migration from one ED to another [1], means they are often referred to different units, one to manage their eating and weight disorder, another to treat their impulsivity and self-harm and yet another to manage comorbid substance misuse. In an attempt to focus treatment, an integrated model of care was developed. We chose to compare mentalisation-based treatment (MBT), which has proved efficacious with patients with BPD [2], with specialist supportive clinical management (SSCM), which has been used with benefit in trials of therapy for anorexia nervosa [3]. We chose MBT on the basis that behaviours associated with EDs, including problems with body image, impulsivity and self-harm, are all symptoms related to vulnerabilities to loss of mentalising. Moreover, MBT is particularly appropriate for testing in eating disordered patients because the latter have been shown to have several features linked to mentalising function, including attachment insecurity [4], problems with emotion regulation [5, 6], disturbances of emotional theory of mind (eTOM) in anorexia nervosa, including recovered patients [7], and unusually distributed mentalising function in bulimia nervosa [8].

Because MBT has not been reported in studies of patients with EDs and because SSCM is designed for patients with anorexia nervosa only, we needed to modify MBT so that it was relevant to participants with EDs, and SSCM so that it could be applied to all EDs. We called the two modified therapies MBT-ED and SSCM-ED.

Our primary objective was to evaluate whether MBT is clinically effective in reducing observer rated symptoms of ED, using an accepted measure, the Eating Disorder Examination, in patients with combined eating and borderline personality disorder symptoms up to 18 months post randomisation compared to SSCM. Secondary objectives included evaluation of the same therapy in reducing symptoms of BPD using a standard assessment [9] and evaluating the health economic implications of the therapy. The full protocol for the study has been published [10].

Recruitment into NOURISHED (Nice OUtcomes for Referrals with Impulsivity, Self Harm and Eating Disorders) was difficult, and maintenance of patients in long-term treatment and follow-up proved problematic. We therefore aim to describe the patterns of recruitment and follow-up, providing estimates of effect size at the follow-up assessments, and to discuss the challenges of this trial.

## Methods

### Study design

This multi-centre study ran across five clinical centres: three NHS Eating Disorder Units and two NHS Personality Disorder Units, located in four clinical sites, three in London, UK and one in Maidstone, Kent, UK.

The design is a single-blind (researchers and statisticians are blind) randomised controlled trial of MBT-ED. MBT-ED consisted of one individual and one group session per week for one year. SSCM-ED comprised one session every 1–4 weeks for 20 to 26 sessions over one year. All trial participants had access to 5 hours of dietetic advice over the course of treatment.

All participants were asked to complete a battery of questionnaires at assessment and at 6, 12 and 18 months follow-up. Because of high drop-out, a further postal assessment was done at 36 months.

### Participants

Patients were considered for participation if they were:

1. Aged 18 years or older
2. Had a Diagnostic and Statistical Manual of Mental Disorders (DSM)-IV [11] diagnosis of an eating disorder
3. Fulfilled either DSM-IV criteria [11] for BPD or had ’BPD symptoms’. The criteria for BPD symptoms were both of the behavioural criteria of DSM-IV:
  - Impulsivity in at least two areas that are potentially self-damaging (e.g. spending, sexual behaviour, substance abuse, reckless driving, binge eating)
  - Recurrent suicidal behaviour or self-mutilating behaviour.

Exclusion criteria were current psychosis based on the Mini International Neuropsychiatric Schedule (MINI) examination, current inpatient or day-patient (attending 3 or more days per week), currently in individual or group psychological therapy, received MBT less than 6 months prior to randomisation, organic brain disease leading to significant cognitive impairment or body mass index (BMI) less than 15 kg/m^2^ (normal range is 18.5–25).

### Recruitment and consent

Participants were recruited from the clinical centres by referral from doctors working in the outpatient services of each centre. Referrals were received by the trial manager, who contacted the potential participant, provided the Participant Information Sheet and then instructed a research assistant to arrange a meeting with the potential participant, obtain informed consent and conduct the initial assessment. All eligible patients were asked to consider participating in the trial, and consent was obtained at least 24 hours after the patient was given the Participant Information Sheet.

### Assessments

Outcome measures were collected pre randomisation (baseline) and at 6, 12 and 18 months post randomisation. The primary outcome was the global score on the Eating Disorder Examination (EDE) at 18 months. The EDE assesses eating disorder psychopathology [12]. It is rated through the use of four subscales (Restraint, Eating, Shape Concern and Weight Concern) and a global score (0–41). Higher scores indicate a greater level of symptomatology.

Other measures used are described as follows:

- The Mini International Neuropsychiatric Schedule (MINI) [13] is a short structured clinical interview for diagnoses of psychiatric disorders according to DSM-IV or International Classification of Diseases (ICD)-10.
- Structured Clinical Interviews for DSM-IV (SCID I, SCID II) are diagnostic instruments used to assess for the presence of Axis I, mental disorders, and Axis II, personality disorders [14].
- Global Assessment of Functioning (GAF) [15] is a numerical scale (1 to 100) used to rate social, occupational and psychological functioning. A higher score is associated with fewer symptoms. The GAF has adequate inter-rater reliability (intraclass correlation 0.81 [15]).
- The Zanarini Rating Scale for Borderline Personality Disorder (ZAN-BPD) [9] is a continuous measure for assessment of DSM-IV borderline psychopathology. Each of the nine criteria for BPD is rated by a trained observer on a 5-point anchored rating scale of 0 to 4, yielding a total score of 0 to 36.
- EuroQol-5D (EQ-5D) is a standardized instrument for use as a measure of health outcome [16].
- General psychopathology was assessed using the Depression, Anxiety, Stress Scale-21 (DASS-21) [17], a 21-item self-report measure that assesses mood state over the past 7 days using a 4-point Likert scale. The total scale is used as a measure of distress; higher scores indicate more symptomatology.
- The Adult Service Use Schedule (AD-SUS) [18] is an interview which was administered by a research worker, covering employment and use of hospital and community services.
- The Big Five Inventory (BFI) [19] is a self-rated, 46-item questionnaire which yields five subscores for personality dimensions: Extraversion, Agreeableness, Conscientiousness, Neuroticism and Openness.

Apart from the MINI and the SCID II, which were only administered at baseline for diagnostic purposes, all instruments were repeated at 6, 12 and 18 months post randomisation.

As the majority of participants were in the normal weight range, we chose not to include body weight or BMI in outcome measures.

Because attendance for follow-up interviews at 18 months was poor (15 out of the original 68), we decided to do a postal questionnaire follow-up at an average of 36 months after randomisation, offering a small financial incentive to participants returning the completed questionnaires. The questionnaires completed at 36 months were the EDE-Q, the DASS and the GAF.

## Safety monitoring

The safety of patients, particularly in view of the possibility of self-harm and physical problems due to the eating disorder, was assured by research and clinical staff at the participating research sites.

### Randomisation, allocation concealment and protection against bias

Due to the nature of the intervention, blinding of participants and therapists was not possible. However, the trial statistician and research workers responsible for the collection of the assessments remained blind to treatment allocation during the trial and primary analyses. At every meeting with participants, research workers reminded them that they were not allowed to know which treatment the participants had received.

Following completion of the baseline questionnaire, patients who consented to take part in the trial were randomly allocated to MBT-ED or SSCM-ED (ratio 1:1) by site via an on-line system based at the King’s College London Clinical Trials Unit, at the Institute of Psychiatry, Psychology and Neuroscience in London. The method of randomisation of participants was block randomisation stratified by BMI (15.0–18.5, 18.6–24.9, >25). Randomly varying block sizes were implemented in order to maintain pre randomisation allocation concealment. The trial manager used the randomisation result to allocate participants to a treatment.

### Intervention

#### Experimental condition: mentalisation-based treatment (MBT-ED)

This treatment was based on the Intensive Outpatient Therapy model [2]. The therapist provided a weekly 50-minute individual MBT-ED session. Participants also attended weekly group MBT-ED for 90 minutes with one or two therapists and four to six patients. Therapy lasted for 12 months, after which patients were reassessed by a member of the trial clinical team and referred for further management if required. Participants assigned to MBT-ED were offered 44 individual sessions and 44 group sessions over the course of 12 months. Thus, the total number of hours offered was 102.7 hours over 12 months.

The Chief Investigator (PR) wrote a manual for the treatment of patients with EDs and BPD using as a basis the already published description of MBT [20]. This approach, MBT-ED, has been summarised [21].

#### Control condition: specialist supportive clinical management for eating disorders (SSCM-ED)

This therapy was based on SSCM as described by McIntosh et al. [3]. SSCM was designed for patients with a diagnosis of anorexia nervosa and has been used in several trials for this patient group [3, 22, 23]. We decided to use SSCM as the basis for the treatment of any eating disorder, reasoning that the key symptoms of EDs in general (namely food restriction, body image disturbance, binge eating, vomiting and laxative abuse) all occur in anorexia nervosa, for which SSCM was designed. We therefore developed an extended form of SSCM and called it SSCM-ED. With the collaboration of Dr McIntosh and Professor Bulik, the SSCM manual was adapted to address other EDs, i.e. ’bulimia nervosa’, ’binge eating disorder’ and ’eating disorder not otherwise specified’. The basic format of SSCM was retained, and this approach includes many of the behavioural elements of cognitive behavioural therapy for eating disorders [24]. Therapists were told that therapy sessions could be curtailed after 30 minutes if appropriate. However, in practice, SSCM-ED sessions lasted 60 minutes. The total number of sessions was 20, with a possibility of up to 6 more sessions at the end if patient, therapist and supervisor agreed. Thus, the total therapy time offered was between 20 and 26 hours over 12 months.

### Users’ involvement 

Two service user consultants (SUCs) and one carer consultant (CC) were recruited to help with the trial. They contributed to the writing of documents such as patient and carer information leaflets and user satisfaction questionnaires, they acted as interviewers on appointment committees and attended telephone conferences for the Trial Steering Committee (TSC). Their contribution was highly valued by the Chief Investigator and other members of the trial team, including the Chair of the TSC. Their hours and transport were paid. In their feedback they suggested that SUCs and CCs should be involved more in future trials.

### Adherence to treatment model 

Adherence to the treatment model was tested by the supervisors. After the trial, seven recorded and transcribed sessions each of MBT-ED (individual therapy, four therapists) and SSCM-ED (seven therapists) were randomly selected and subjected to adherence rating. For MBT-ED a trained research assistant (AR) rated the sessions using the MBT Adherence and Competence Scale (MBT-ACS) [25] with a 7-point scale in which 4 represents adequate therapy. The transcriptions were rescored by an experienced rater (FS) whose adjustments were accepted. There is no published adherence scale for SSCM. PR developed an approach which counts the number of times a therapist followed the manual and the number of times the therapist did not follow the manual. This led to an overall score of adherence, using a 7-point scale as in the MBT-ACS, with a score of 4 representing adequate therapy. Seven randomly chosen transcripts were scored by an independent observer (AR), and the transcripts were read and rescored by a senior author (PR).

### Statistical analysis

#### Power calculation

The sample size calculation was based on the mean difference in global EDE scores at 18 months post randomisation. We utilised an effect size of 1.07 based on a minimal clinically important difference of 1.5 points with a pooled standard deviation of 1.4. An overall sample size of 140 would provide more than 90 % power to detect an effect size of *d* = 1.07 using an analysis of covariance with two-sided 5 % significance tests. This calculation included inflation for 25 % attrition; made the conservative assumption of no correlation between baseline and follow-up; and allowed for clustering of group treatment in the MBT-ED arm. We assumed an average group size of 10 and an intraclass correlation between EDE scores of 0.07, which resulted in a design effect of 1.63.

### Analysis

The statistical analysis plan was finalised and approved by the TSC. A value of *p* < 0.05 was regarded as significant for all analyses and based on the intention-to-treat sample. We summarised continuous variables as mean (SD) and categorical variables as *n* (%). Unadjusted effect sizes (Cohen’s *d*) were calculated at 6, 12 and 18 months post randomisation for all outcomes. Not all questionnaires and interviews anticipated in the protocol [10] were included in this analysis. Some were excluded because the small numbers remaining at follow-up did not justify statistical analysis of outcome over time, and others (the Object Relations Inventory (ORI), treatment adherence, Reading the Mind in the Eyes test and the Reflective Functioning Questionnaire (RFQ)) will be described elsewhere (for references see [10]).

EDE and ZAN-BPD outcomes were analysed using linear mixed models. The dependent variable was the outcome, with trial arm, baseline value of the variable under investigation, randomisation stratifier (BMI) and time (6, 12, 18 and 36 months where available) as covariates. We included a time by treatment interaction term to allow estimates at the individual time points to be summarised.

The models incorporated random intercepts for participants and an additional random effect for therapists. This allowed for correlations in outcomes related to treatment from repeated measures from the same individual and by therapist, respectively.

Model assumptions were checked by use of diagnostic plots. Due to the small number of follow-up measures, the stability of significant results was checked by bias corrected confidence intervals, bootstrapped with 1000 repetitions. We did modelling with the assumption that data were missing at random, and included predictors of missing data (neuroticism and smoking status) in the modelling. We used a logistic model to assess predictors of missing data (examination of all baseline clinical and demographic variables). Due to the low number of follow-up assessments, no moderation or mediation analyses were completed.

We completed exploratory pre/post analyses also utilising linear mixed modelling. Assessments at baseline were included as part of the outcome, and time as a covariate included 0 (baseline) to allow estimates of change to be summarised in the treatment groups and overall. We summarised results from the main outcomes EDE and ZAN-BPD at 6, 12, 18 and 36 months post randomisation with two-sided 95 % CIs. All analyses were done with Stata version 13.0 [26].

### Economic evaluation

The economic evaluation took a National Health Service (NHS)/Personal Social Services (PSS) perspective as recommended by the National Institute for Health and Care Excellence (NICE), including the cost of all hospital and community health and social care services. Resource use information was collected using a modified version of the Adult Service Use Schedule (AD-SUS). Resource use data were combined with appropriate national unit costs to calculate the total cost of the intervention and control groups. The costs of the MBT-ED were directly calculated from salaries using a microcosting approach [27]. Differences in mean total costs between groups were compared using the standard *t* test with ordinary least squares regression used for adjusted analyses and the validity of results confirmed using bootstrapping [28].

## Results

### Results relevant to the conduct of the trial

#### Recruitment of participants

The power calculation indicated that 140 participants would be required in total. Recruitment, which occurred between July 2011 and November 2012, was brisk at the beginning and then flattened while the recruited participants were treated. Recruitment picked up again when a new cohort of participants could be treated. The length of the study was dictated by the funding available.

The factors which affected recruitment were (1) delays in recruiting research workers due to funding restrictions, (2) availability of trained therapists, especially for MBT-ED, and (3) agreement of patients to participate in the study.

**Fig. 1:**
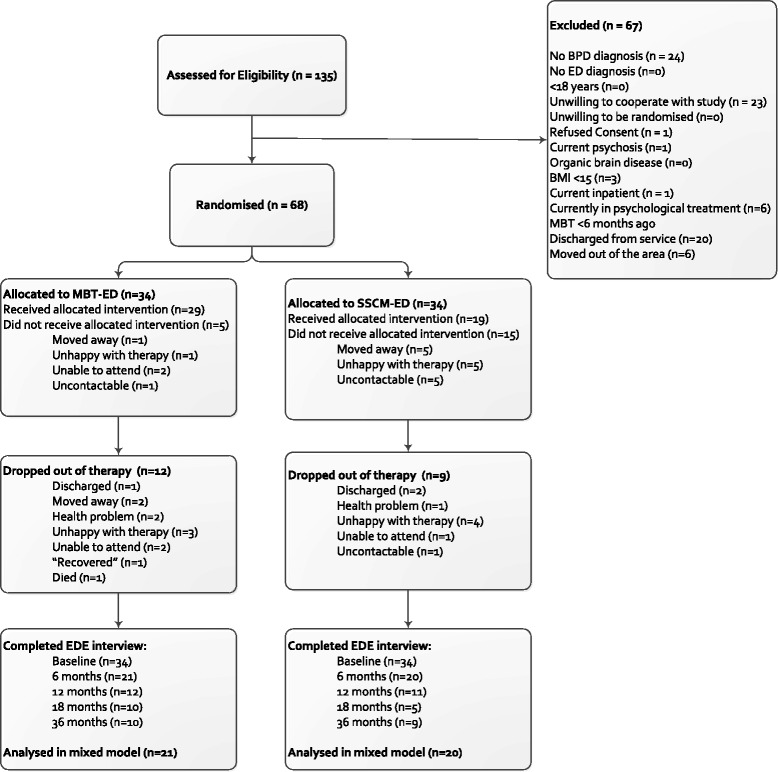
Consolidated Standards Of Reporting Trials (CONSORT) diagram showing the participants remaining at each stage of the trial

During the two-and-a-half-year recruitment period, a total of 135 patients were screened for eligibility, and 68 participants were randomised in the trial. Reasons for ineligibility are given in Fig. 1.

### Baseline data

#### Demographics

Demographics and clinical characteristics are summarised in Table 1. In particular, our participant population was 92.7 % female, reflecting gender differences in attendance of eating disorder clinics reported elsewhere [29].

**Table 1:** Baseline characteristics of the sample

		MBT-ED	SSCM-ED	Total
		(*N* = 34)	(*N* = 34)	(*N* = 68)
Demographics	Age, years (mean [SD])	31.2 (9.8)	30.8 (10.0)	31.1 (9.9)
	Gender, female (*n* [%])	32 (94.1 %)	31 (91.2 %)	63 (92.7 %)
	Ethnicity, white (*n* [%])	28 (82.4 %)	29 (85.3 %)	57 (83.8 %)
	Ever smoked (*n* [%])	22 (64.7 %)	25 (73.5 %)	47 (69.1 %)
	Years in education (mean [SD])	19.9 (3.5)	19.0 (3.0)	19.4 (3.3)
Clinical	Weight (kg) (mean [SD])	69.41 (28.2)	63.16 (13.4)	66.23 (22.0)
	BMI (mean, SD)	25.4 (11.4)	22.9 (4.5)	24.1 (8.6)
	BMI 15–18.5 (*n* [%])	6 (17.7 %)	5 (14.7 %)	11 (16.2 %)
	BMI 18.6–24.9 (*n* [%])	18 (52.9 %)	18 (52.9 %)	36 (52.9 %)
	BMI >25 (*n* [%])	10 (29.4 %)	11 (32.4 %)	21 (30.9 %)
	Diagnosis anorexia nervosa (*n* [%])	2 (5.9 %)	2 (5.9 %)	4 (5.9 %)
	Diagnosis bulimia nervosa (*n* [%])	22 (64.7 %)	21 (61.8 %)	43 (63.2 %)
	Diagnosis binge eating disorder (*n* [%])	2 (5.9 %)	-	2 (2.9 %)
	Diagnosis EDNOS (*n* [%])	8 (23.5 %)	11 (32.4 %)	19 (27.9 %)
	EDE global (mean [SD])	4.40 (1.0)	4.23 (0.8)	4.31 (0.9)
	EDE Eating Concern (mean [SD])	3.54 (1.21)	3.47 (1.14)	3.51 (1.17)
	EDE Shape Concern (mean [SD])	5.09 (0.92)	4.83 (0.92)	4.96 (0.92)
	EDE Weight Concern (mean [SD])	4.71 (1.19)	4.56 (1.36)	4.64 (1.27)
	ZAN-BPD (mean [SD])	16.12 (6.3)	16.74 (6.0)	16.43 (6.1)
	DASS-21 (mean [SD])	26.3 (8.3)	29.18 (9.1)	27.76 (8.8)
	EuroQol VAS (mean [SD])^a^	48.09 (20.9)	50.56 (19.9)	49.32 (20.3)
	GAF (mean [SD])^a^	47.32 (6.3)	49.12 (6.5)	48.22 (6.4)

*BMI* body mass index, *EDNOS* eating disorder not otherwise specified, *EDE* Eating Disorder Examination, *DASS-21* Depression, Anxiety, Stress Scale, *GAF* Global Assessment of Functioning, *ZAN-BPD* Zanarini Rating Scale for Borderline Personality Disorder

^a^Indicates a higher value is a better outcome

The proportion of participants from ethnic minorities was less than expected (14.7 %) considering that, for London boroughs local to the main recruitment site, census data [30] suggest some 42 % of residents are of non-White ethnicity. The two treatment arms were reasonably balanced on all baseline characteristics.

## Clinical information

General ability to function, as measured by the GAF, was affected in all but one participant to at least a moderate extent; 57.4 % of participants showed at least ’serious’ impairment on the scale.

The MINI [13] revealed a broad range of psychiatric disorders with 88.2 % fulfilling criteria for affective disorder and 93.3 % assessed as having a high risk of suicide. The SCID-II showed that 92.1 % of the participants fulfilled criteria for a personality disorder: 76 % had BPD and 16 % depressive or avoidant PD. The mean scores on the DASS in our patients were all in the Extremely Severe ranges, which are more than 14 for depression, more than 10 for anxiety and more than 17 for stress.

In summary, the 68 participants recruited for the NOURISHED study had very high scores on measures of eating disorder, general psychiatric symptoms and BPD descriptive criteria. They mostly suffered from bulimia nervosa (BN) or eating disorder not otherwise specified (EDNOS), and most had BPD. None of the diagnoses was significantly more represented in one treatment condition compared to another.

Only 7 patients had a BMI less than 18.5, and in 6 participants the BMI was more than 30, leaving the majority, 55 participants, in the range 18.5–30.

### Treatment information

#### Therapy training and supervision

MBT-ED therapists in the study fulfilled the following criteria. (1) They had received basic mental health training (such as Mental Health Nursing, Psychotherapy, Psychiatry, Clinical Psychology or Occupational Therapy). (2) They had completed a recognised basic training in MBT. (3) They had attended a one-day MBT-ED course provided by senior members of the research team (Prof AB, PR). (4) They had treated a patient with MBT or co-run an MBT group under qualified supervision for at least 6 months. During the trial, therapists provided recordings of group and individual MBT-ED sessions and attended regular supervision, including review of the recordings.

#### SSCM-ED

Staff wishing to provide SSCM-ED as part of the NOURISHED study needed to fulfil the following criteria: (1) working in a post in which patients with EDs form a substantial part of the caseload, (2) having worked with patients with EDs for at least 6 months, (3) having attended a one-day training in SSCM-ED by a senior member of the research team (PR), (4) attending supervision by an experienced supervisor at least monthly, providing session recordings, (5) providing random session recordings to be reviewed by an experienced supervisor. During the trial, as for MBT-ED, regular supervision was attended and therapy recordings provided.

The two trial therapies were set up at all four research sites together with supervision, according to the protocol. Therapy provision presented significant challenges. The need for training and experience delayed recruitment of therapists, particularly for MBT-ED. There was significant staff turnover amongst therapists, two Principal Investigators left their posts and clinical services were altered by management, compromising provision of trial interventions. Three MBT-ED therapists and three SSCM-ED therapists (out of 28 therapists in total) left their posts before therapy with their patients had ended. The resulting delays and disruptions accounted for much of the shortfall in recruitment and in retention within therapy.

### Treatment compliance and drop-out from therapy

Twenty participants dropped out before attending any sessions; a further 18 dropped out after beginning therapy. Twenty-nine participants dropped out after attending less than 50 % of sessions offered, and 8 dropped out after attending more than 50 %.

We set a level of 50 % attendance [10] to indicate compliance. That level was achieved by 47.1 % in the MBT-ED arm and 37.1 % in the SSCM-ED arm. The compliance with research assessment follow-up is presented in the CONSORT diagram (Fig. 1).

We analysed the baseline data for factors that might predict drop-out. Only two factors emerged: Neuroticism on the Big Five Inventory of Personality [19] and smoking status, current smokers being more likely to drop out of the study. Neuroticism was entered into a correlation matrix (Pearson’s *r*) and correlated positively with the Stress and Anxiety scales of the DASS (*r* = 0.54, *p* = 0.000, *r* = 0.47, *p* = 0.000) and negatively with the measures of quality of life (Physical *r* = −0.48, *p* = 0.000, Psychological *r* = 0.34, *p* = 0.004) [16]. Of those participants allocated to SSCM-ED, more (15 participants, 44 %) failed to start treatment compared to those allocated to MBT-ED (5 participants, 15 %). This difference is significant (chi-squared = 7.08, *p* = 0.008, Fisher’s exact test: *p* = 0.015).

#### Reasons given for dropping out of therapy

Of the 53 participants who dropped out by 18 months, reasons were provided by 32 and a further 7 did not respond to repeated offers of therapy appointments. One participant died. Five participants randomised to MBT-ED cited therapy-related reasons for dropping out, compared to 9 randomised to SSCM-ED. Reasons included disappointment at being randomised to either MBT-ED or SSCM-ED (with no difference between the therapies); distress over having to wait for therapy; feeling that the therapy was not helping. Fourteen participants (9 MBT-ED and 5 SSCM-ED) had moved away or were unable to attend due to work commitments. There was no significant difference in reasons for drop-out between therapies and no significant difference in drop-out rate between the four clinical sites.

### Adherence with treatment model 

For MBT-ED, reports from all supervisors were satisfactory. This was supported by the results of the MBT-ACS used in seven randomly selected individual sessions. The adherence scores (with number of sessions scoring that level in brackets) were 7 (1), 6 (3), 4 (3). Competence scores were identical to adherence scores. Karterud et al. [25] give a rating of 4 as acceptable for MBT therapists.

For SSCM-ED, reports from supervisors were also satisfactory. The agreed SSCM-ED adherence scale results on seven randomly selected sessions (seven different therapists) were adherence 7 (2), 6 (1), 5 (1), 4 (3).

### Adverse events during the trial

Ten participants were reported as having had adverse events during the trial, including one death, in a patient receiving MBT-ED. One participant had multiple events, including self-harm and suicidal threats.

All events were followed up by the Chief Investigator and reported to the Trial Oversight Committees. Internal and external investigations into the death found no evidence of any act or omission on the part of the treatment team which would have directly caused or contributed to her death, and the final verdict of the coroner was ’Unexplained Sudden Adult Death’.

#### Drop-out from research assessments

The CONSORT diagram (Fig. 1) demonstrates the very high level of drop-out from follow-up assessment seen in this study. Research workers were unable to contact four participants, and some (15 %) had moved away. For others, the participant would agree to attend the 4-hour reassessment and then not appear, sometimes failing to attend a subsequent appointment that was offered. Research workers and the Chief Investigator used every possible medium to contact participants. In the final year of the study, some questionnaires were omitted, reducing the time of the assessment to 1–2 hours, but this did not seem to affect attendance. At 36 months, participants were sent an envelope of questionnaires as a long-term follow-up assessment and offered a £10 voucher for returning them, as well as the chance of winning a £200 prize. Only 19 participants returned their questionnaires. The numbers shown in Fig. 1 demonstrate no statistical difference in drop-out from research assessments between the two arms of the study.

Baseline variables that showed association with missing data at the follow-up assessments were neuroticism and smoking behaviours. High scoring neuroticism and smoking may be associated with higher anxiety, and more anxious participants may have found the trial too stressful. We also found evidence that higher baseline BMI is associated with higher levels of drop-out.

### Results relevant to testing the research hypotheses

#### Treatment outcomes

Table 2 shows the results of the mixed modelling adjusted for baseline score, BMI and predictors of missingness. All data up to 36 month outcomes were included in the modelling. Due to the small sample sizes involved in these analyses, all inference estimates must be interpreted as exploratory.

**Table 2:** Outcomes for the EDE scales and ZAN-BPD global

Outcome	6 months		12 months		18 months		36 months	
	MBT-ED	SSCM-ED	MBT-ED	SSCM-ED	MBT-ED	SSCM-ED	MBT-ED	SSCM-ED
	(*n* = 21)	(*n* = 20)	(*n* = 12)	(*n* = 11)	(*n* = 10)	(*n* = 5)	(*n* = 10)	(*n* = 9)
Summaries (Mean [SD])								
EDE global	3.86 (1.34)	4.12 (0.95)	3.75 (1.81)	3.78 (0.95)	3.35 (1.80)	3.73 (1.35)	3.76 (1.54)	4.13 (1.15)
EDE Eating Concern	3.09 (1.55)	3.28 (1.63)	3.22 (1.88)	2.45 (1.44)	2.78 (2.02)	3.08 (1.75)	3.11 (1.69)	3.23 (1.67)
EDE Shape Concern	4.50 (1.28)	4.77 (0.96)	4.37 (1.84)	4.95 (0.65)	3.81 (1.85)	4.78 (0.90)	4.34 (1.54)	4.88 (0.97)
EDE Weight Concern	4.46 (1.27)	4.58 (1.09)	3.99 (2.03)	4.76 (0.70)	3.72 (1.82)	4.76 (0.99)	4.17 (1.60)	4.74 (1.04)
EDE restraint	3.38 (1.79)	3.84 (1.00)	3.44 (2.14)	2.95 (1.75)	3.08 (2.40)	2.32 (2.20)	3.42 (1.99)	3.64 (1.68)
ZAN-BPD global	11.76 (7.42)	13.25 (8.69)	9.64 (7.41)	9.27 (7.39)	7.1 (6.19)	10 (7.97)		
Inference	Mean difference from SSCM	*p* value	Mean difference from SSCM	*p* value	Mean difference from SSCM	*p* value	Mean difference from SSCM	*p* value
	(SE)	(95 % CI)	(SE)	(95 % CI)	(SE)	(95 % CI)	(SE)	(95 % CI)
EDE global	−0.35 (−0.34)	0.31 (−1.02 to 0.33)	−0.43 (−0.31)	0.16 (−1.03 to 0.17)	−0.51 (−0.31)	0.10 (−1.12 to 0.10)	−0.75 (−0.53)	0.16 (−1.79 to 0.29)
EDE Eating Concern	−0.02 (−0.36)	0.97 (−0.79 to 0.76)	−0.10 (−0.36)	0.78 (−0.80 to 0.60)	−0.19 (−0.36)	0.61 (−0.90 to 0.52)	−0.44 (−0.57)	0.44 (−1.56 to 0.68)
EDE Shape Concern	−0.67 (−0.34)	0.05 (−1.33 to 0.01)	−0.66 (−0.30)	0.03 (−1.25 to −0.07)	−0.66 (−0.30)	0.03 (−1.25 to − 0.07)	−0.64 (−0.51)	0.20 (−1.64 to 0.35)
EDE Weight Concern	−0.47 (−0.35)	0.18 (−1.17 to 0.22)	−0.56 (−0.32)	0.07 (−1.19 to 0.05)	−0.66 (−0.32)	0.04 (−1.29 to − 0.04)	−0.95 (−0.52)	0.07 (−1.96 to 0.06)
EDE restraint	−0.26 (−0.49)	0.60 (−1.22 to 0.70)	−0.36 (−0.43)	0.41 (−1.20 to 0.48)	−0.45 (−0.44)	0.30 (−1.31 to 0.40)	−0.75 (−0.76)	0.33 (−2.24 to 0.75)
ZAN-BPD global	−2.41 (−1.87)	0.20 (−6.07 to 1.25)	−2.50 (−1.84)	0.18 (−6.11 to 1.11)	−2.59 (−2.49)	0.30 (−7.47 to 2.29)		

Comparisons of the differences were made at 6, 12, 18 and 36 months from the final adjusted linear mixed model

In terms of the primary outcome, treatment differences indicated that while there was no significant difference between the two interventions in global EDE score, two of the subscales showed evidence of significantly lower scores in the MBT-ED group at 12 and 18 months. These were, at 18 months, Shape Concern (adjusted mean difference = −0.7, *p* = 0.029, 95 % CI = −1.25 to −0.07) and Weight Concern (adjusted mean difference = −0.7, *p* = 0.037, 95 % CI = −1.29 to −0.04).

The global ZAN-BPD score did not differ significantly between groups at either 6 (*p* = 0.198), 12 (*p* = 0.175) or 18 (*p* = 0.298) months.

There were no significant effects of treatment on any of the EDE subscales at 36 months. Additional file 1: Figure S1 shows the treatment profile plots over the course of the NOURISHED study for the EDE and ZAN-BPD scales.

#### Standardised effect sizes

Table 3 shows the unadjusted effect sizes and 95 % confidence intervals for the trial outcomes. The largest standardised effect sizes were seen at 18 months for the EDE and ZAN-BPD scales, ranging from Cohen’s *d* = −0.16 (95 % CI −1.23 to 0.92) for EDE Eating Concern to −0.60 (95 % CI −1.68 to 0.51) for EDE Shape Concern. However measures of functioning and quality of life showed the largest difference at 12 months. The confidence intervals indicate the variability in the data from the small sample sizes.

**Table 3:** Unadjusted effect sizes between MBT-ED and SSCM-ED with 95 % confidence intervals

	6 months	12 months	18 months
	Cohen’s *d* (95 % CI)	Cohen’s *d* (95 % CI)	Cohen’s *d* (95 % CI)
Eating Disorder Examination			
Eating Concern	−0.12 (−0.74 to 0.49)	0.45 (−0.36 to 1.24)	−0.16 (−1.23 to 0.92)
Shape Concern	−0.24 (−0.85 to 0.38)	−0.40 (−1.20 to 0.40)	−0.60 (−1.68 to 0.51)
Weight Concern	−0.10 (−0.72 to 0.51)	−0.49 (−1.29 to 0.32)	−0.65 (−1.74 to 0.47)
Restraint	−0.31 (−0.93 to 0.31)	0.25 (−0.55 to 1.04)	0.33 (−0.76 to 1.40)
Global	−0.22 (−0.84 to 0.39)	−0.02 (−0.81 to 0.77)	−0.23 (−1.30 to 0.85)
Zanarini Scale			
Global score	−0.19 (−0.78 to 0.43)	0.05 (−0.74 to 0.84)	−0.43 (−1.51 to 0.67)
Depression Anxiety Stress Scale			
Stress	−0.78 (−1.41 to − 0.14)	−0.21 (−1.02 to 0.62)	−0.11 (−1.20 to 0.99)
Anxiety	−0.07 (−0.69 to 0.56)	0.26 (−0.54 to 1.05)	−0.04 (−1.13 to 1.06)
Depression	−0.34 (−0.95 to 0.28)	0.19 (−0.60 to 0.98)	0.51 (−0.62 to 1.61)
World Health Organisation Quality of Life			
Physical health	−0.15 (−0.76 to 0.46)	−0.14 (−0.93 to 0.65)	−1.18 (−2.35 to 0.03)
Psychological Health	−0.07 (−0.68 to 0.55)	−0.13 (−0.92 to 0.66)	−0.41 (−1.51 to 0.71)
Social relationships	0.33 (−0.29 to 0.94)	−0.10 (−0.89 to 0.69)	−0.06 (−1.15 to 1.04)
Environment	−0.45 (−1.07 to 0.17)	−0.37 (−1.17 to 0.43)	−0.28 (−1.37 to 0.83)
Global Assessment of Functioning			
Total score	−0.27 (−0.88 to 0.35)	−0.07 (−0.86 to 0.72)	−0.44 (−1.52 to 0.65)

#### Exploratory changes over time

Linear mixed models including the baseline assessments as part of the dependent variable were used to evaluate changes in the scores over the course of the study within trial arms. There was evidence of improvements in EDE global scores at 6, 12 and 18 months from baseline in the MBT-ED trial arm. At 18 months, MBT-ED showed a −1.2 point reduction from baseline (95 % CI −1.81 to −0.56, *p* < 0.001). However, the SSCM-ED showed less improvement on the global scale (18 month change from baseline −0.5, 95 % CI −1.38 to 0.29, *p* = 0.200).

This trend did not extend to the 36 month time point for either treatment. Profile plots show a slight increase in EDE scores at 36 months. Please note the low sample size (*n* = 19) at this time point and probable reporting bias.

**Fig. 2:**
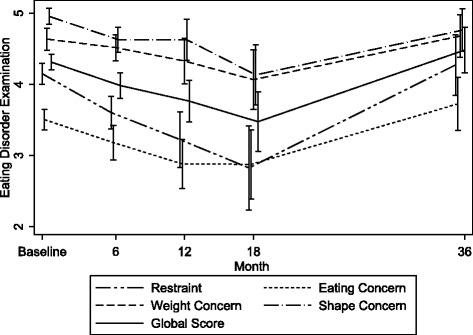
Unadjusted mean EDE scores for the whole trial sample for all post randomisation time points and baseline. Lowest score is best. Error bars show standard error

An examination of the whole group, irrespective of treatment, showed that global EDE score, Shape Concern and Weight Concern reduced up to the 18 month point, but not beyond (Fig. 2).

There was a fall in global ZAN-BPD scale for both trial arms at 6, 12 and 18 months. The largest decrease was seen again at 18 months: MBT-ED mean change from baseline −8.8 (95 % CI −12.68 to −4.95, *p* < 0.001), SSCM-ED mean change −7.5 (95 % CI −12.49 to −2.55, *p* = 0.003).

#### Health economic analysis

Conclusions regarding service use are tentative due to the very small numbers with full data. Use of community services appears to be higher in the SSCM-ED group, but admissions were more likely in the MBT-ED group.

Similarly, it is difficult to use the cost data to draw conclusions. Over 18 months follow-up, costs in the MBT-ED group are, on average, more than £3000 higher than in the SSCM-ED group. It appears that this is predominantly due to the higher cost of the MBT intervention. The difference in cost was not statistically significant.

EQ-5D scores are generally similar between randomised groups, and there is a slightly higher quality-adjusted life years (QALY) score in the MBT-ED group, but this may be due to the small numbers for whom it was possible to calculate a QALY.

## Discussion

### Main results of the trial

The NOURISHED project is a study of two novel therapies, MBT-ED and SSCM-ED, in patients with EDs and symptoms of BPD. This study has been reported in accordance with CONSORT guidelines (see Additional file 2). An unusually high drop-out rate makes any conclusions about effectiveness tentative. The study was well designed and prepared by a large group of collaborators with the active support of a highly regarded Clinical Trials Unit. In spite of this, recruitment was difficult and maintenance of patients in long-term treatment and follow-up problematic. The most significant result of the study was to demonstrate the difficulties encountered in recruiting, treating and retaining participants in the trial in a very complex population.

Participants who stayed in the trial, irrespective of treatment, showed significant clinical improvement in both ED and BPD symptoms, although, because of the drop-out rate, the significance of this finding remains uncertain. Treatment trials for bulimia nervosa using CBT report the following changes in EDE global score between baseline and end of treatment: 1.1 [31], 1.6 [29] and 1.9 [32] using CBT-E, and 1.4 [33] using guided self-help. In our study the EDE global score change at 18 months was 1.2 for MBT-ED and 0.5 for SSCM-ED. Treatment trials for BPD which use the ZAN-BPD are few, but McMain et al. [34] report a reduction of 7.6 points using dialectical behaviour therapy. In our study the mean reduction in ZAN-BPD for both arms of the trial was 8.8 at 18 months.

Two of the EDE subscales, Shape Concern and Weight Concern, showed significantly lower scores for the MBT-ED compared to SSCM-ED at 18 months — differences that were also detectable at 12 months. This result needs to be interpreted with caution, due to drop-outs. If reliable, it could indicate that the treatment model of MBT-ED may have been superior to SSCM-ED. However, it could be an effect of therapy ’dose’, as MBT-ED provided four to five times the number of treatment hours compared to SSCM-ED. In the study, participants allocated to MBT-ED did have more therapy hours than those allocated to SSCM-ED, with 47.1 % of the former attending at least 50 % of individual and group sessions, compared to 37.1 % of the latter.

With regard to feasibility, any future trial must take into account the following:

1. Training for MBT-ED introduced significant delay and should precede the beginning of the trial so that adequate numbers of therapists are available.
2. There was a significant reduction in numbers of participants returning for follow-up assessment.

### Drop-out from the trial

Drop-out can refer to withdrawal from therapy or failure to attend research assessments. The proportion of participants not meeting our criterion of 50 % attendance at therapy was 52.9 % for MBT-ED and 62.9 % for SSCM-ED (a non-significant difference). Regarding research assessments, the CONSORT diagram (Fig. 1) shows a steady decline in attendance with 70.6 % non-attendance in the MBT-ED group at 18 months, compared to 85.3 % for SSCM-ED, also a non-significant difference. Hence, there was evidence that participants withdrew from both therapy and research assessment. The causes are not immediately apparent. We found evidence that participants who scored highly on the neuroticism scale of the BFI and those who were current smokers were more likely to drop out. This may suggest that more anxious participants may have found the trial too stressful. We were aware that some participants (15 %) had moved to different parts of the country. The EDE showed that the population was of high severity, with a mean level of EDE global score of 4.35 (SD 0.94, *n* = 68). Compared to a population of patients with bulimia nervosa [29] in which the global EDQ score was 3.79, our patients scored substantially higher. The ZAN-BPD results (mean 16.12 (SD 6.33) 95 % CI 14.62–17.62) were somewhat higher than the mean found in patients with BPD reported by Zanarini et al. [35], in which study subjects had a mean total score of around 11.5. The instability of participants with BPD may be a significant factor contributing to the high drop-out from treatment.

In previous comparable trials, drop-out varies. There is no previous report of an RCT for participants with both EDs and BPD. However, in trials for borderline participants, drop-out rates, albeit variously defined, are reported as 50 % drop-out from therapy and 17 % loss to follow-up [36], 39 % drop-out from therapy and assessment [37], 43 % drop-out, including 19 % before treatment started [38] and 26 % drop-out [2]. For RCTs with participants suffering from EDs, the drop-out rate ranges from 15–70 % [39, 40]. Both EDs and BPD have serious physical, psychological and social morbidity, reflected in the very high baseline levels of eating disorder, borderline and general psychiatric disturbance that we found. It is quite likely that the combination of the two sets of conditions resulted in a level of overall disturbance which affected compliance and increased drop-out.

Participants allocated to SSCM-ED were significantly more likely to drop out before the start of therapy than those allocated to MBT-ED. However, participants who dropped out at different times during therapy did not cite dissatisfaction with therapy in one approach significantly more than the other. Walsh et al. [41], in a study of guided self-help and medication in bulimia nervosa, found an overall drop-out rate of 70 %, amongst whom one-third cited problems with therapy as the reason, compared to one-fifth in our study. In a study of cognitive behaviour therapy and medication in anorexia nervosa [40], 46 % of participants dropped out and a further 17 % were withdrawn due to treatment failure. The only correlate of drop-out found in that study was high self-esteem, which was associated with fewer drop-outs. In a study of CBT and dietary counselling in anorexia nervosa [42], all ten participants in the Dietary Counselling group dropped out of treatment, compared to just one in the CBT group. The reasons for the drop-out are uncertain, but the result was that dietary counselling alone was not regarded as a useful treatment on its own in anorexia nervosa, a view also reflected in the NICE guidelines for eating disorders [43].

Other difficulties encountered in the present study included service and staff changes which affected the trial and which can be expected in an NHS service. However, they were particularly severe in our study, with emigration of two Principal Investigators and limitations on recruitment and treatment capacity due to service changes.

### Positive outcomes of the trial

Two new forms of therapy for patients with EDs were developed. MBT was adapted for use in an eating disordered population (MBT-ED), and this has led to the implementation of MBT-ED in the clinical services taking part in the trial.

SSCM-ED was expanded from the original therapy designed for patients with anorexia nervosa. SSCM-ED can be used for any eating disorder, and this has also been implemented in the clinical services participating in the trial.

The clinical field of EDs has, therefore, benefited from the trial, and as more therapists become competent in the two approaches, a larger randomised controlled trial will be more feasible.

We were fortunate to have a group of users and carers as part of the research team. Their main contribution was in the design of the study and the information sheets, their participation in Trial Oversight Committees and their help on research staff appointment committees. However, in spite of these important contributions, they felt under-used in the trial, and this issue should be reviewed in advance of any future trial. Participants knew that they could see one of the user consultants, and families were told that they could see the carer consultant if they had concerns, but this facility was not used.

### Limitations

Developing two new versions of established therapies and testing them in a multi-centre RCT in a population of high morbidity and complexity within 3–4 years was problematic. The therapies are now developed with some indication of good clinical outcomes, so the challenge of a new study would be to maintain participants in the research for at least 18 months. Craig et al. [44] clearly advise extensive piloting and preparation before embarking on a full RCT, and, with the experience of this study, we fully endorse that advice.

The problem raised by the present study is how to maintain a group of patients with very challenging symptoms who are increasingly seen in clinical practice in a research trial for long enough to determine efficacy. In the present study, the Chief Investigator (PR) was not directing the overall clinical management of the participants. This is in contrast to a randomised controlled trial of MBT in BPD [45] in which treatment and research were carried out in a single psychotherapy unit, directed by the Chief Investigator. In that study the drop-out from therapy was 12 %, and the different model of clinical management may have contributed to that low rate of drop-out.

## Conclusion

Over the first 18 months, in participants remaining in the study, there were improvements in ED and BPD symptoms in the whole group under study, and MBT-ED seemed to be more effective in addressing body image disturbance in EDs complicated by BPD.

The best outcome for patients with bulimia nervosa, the condition experienced by most of our participants, is associated with cognitive behaviour therapy [24]. However, therapy for patients with both EDs and BPD has not been evaluated in a randomised trial, and there are indications that this group would have a less satisfactory clinical outcome [1]. We suggest that a further feasibility study be mounted in order to establish how MBT-ED, perhaps with a comparison therapy, could be provided to patients with both Eating disorders and borderline personality disorder symptoms in a way that is acceptable to those patients.

## Additional files

Additional file 1: Figure S1. Unadjusted mean EDE and ZAN score by treatment group for all post randomisation time points and baseline (time 0). Lowest score if best. Error bars show 95 % CI. *EDE* Eating Disorder Examination, *ZAN* Zanarini Rating Scale. (DOCX 74 kb)
Additional file 2: CONSORT 2010 checklist. (DOCX 209 kb)

## Abbreviations

AD-SUS: Adult Service Use Schedule
BFI: Big Five Inventory
BMI: Body mass index
BPD: Borderline personality disorder
CC: Carer consultant
DASS: Depression, Anxiety, Stress Scale
DSM IV: Diagnostic and Statistical Manual of Mmental Disorders version IV
EDE: Eating Disorder Examination
EDE-Q: Eating Disorder Examination: Questionnaire version
EQ-5D: EuroQol (5 dimensions)
GAF: Global Assessment of Functioning
MBT: Mentalisation-based treatment
MBT-ED: Mentalisation-based treatment: modified for eating disorders
MINI: Mini International Neuropsychiatric Schedule
NOURISHED: Nice OUtcomes for Referrals with Impulsivity, Self Harm and Eating Disorders
ORI: Object Relations Inventory
QALY: Quality-adjusted life years
RCT: Randomised controlled trial
RFQ: Reflective Functioning Questionnaire
SCID: Structured Clinical Interview for DSM-IV
SSCM: Specialist supportive clinical management
SSCM-ED: Specialist supportive clinical management: modified for all eating disorders
SUC: Service user consultant
TSC: Trial Steering Committee
ZAN-BPD: Zanarini Rating Scale for Borderline Personality Disorder
