# Supplementary material for: The NOURISHED randomised controlled trial comparing mentalisation-based treatment for eating disorders (MBT-ED) with specialist supportive clinical management (SSCM-ED) for patients with eating disorders and symptoms of borderline personality disorder
Source: Trials. 2016 Nov 17;17:549. doi: 10.1186/s13063-016-1606-8 (PMC5114835; doi:10.1186/s13063-016-1606-8)
Supplement: Additional file 1: Figure S1. — Unadjusted mean EDE and ZAN score by treatment group for all post randomisation time points and baseline (time 0). Lowest score if best. Error bars show 95 % CI. EDE Eating Disorder Examination, ZAN Zanarini Rating Scale. (DOCX 74 kb) [file 13063_2016_1606_MOESM1_ESM.docx]

Figure 1:

Unadjusted mean EDE and ZAN score by treatment group for all post randomisation time points and baseline (time 0).

Lowest score if best. Error bars show 95% CI.

EDE = Eating Disorder Examination

ZAN = Zanarini Rating Scale
